# The Impact of Antimicrobial Resistance in Cystic Fibrosis

**DOI:** 10.3390/jcm13061711

**Published:** 2024-03-16

**Authors:** Antonio Vitiello, Francesco Blasi, Michela Sabbatucci, Andrea Zovi, Francesco Miele, Annarita Ponzo, Roberto Langella, Mariarosaria Boccellino

**Affiliations:** 1 Ministry of Health, Viale Giorgio Ribotta 5, 00144 Rome, Italy; avitiello@hotmail.it; 2 Respiratory Unit and Cystic Fibrosis Center, Fondazione IRCCS Ca’ Granda Ospedale Maggiore Policlinico, 20122 Milan, Italy; francesco.blasi@unimi.it; 3 Department Infectious Diseases, Italian National Institute of Health, 00161 Rome, Italy; 4 General Surgery Unit, University of Campania “Luigi Vanvitelli”, 80138 Naples, Italy; francesco.miele@policliniconapoli.it; 5 Biology Department, University of Pavia, 27100 Pavia, Italy; annari.ponzo@gmail.com; 6 Italian Society of Hospital Pharmacy (SIFO), SIFO Secretariat of the Lombardy Region, Carlo Farini Street 81, 20159 Milan, Italy; 7 Department of Precision Medicine, University of Campania “Luigi Vanvitelli”, 81100 Naples, Italy; mariarosaria.boccellino@unicampania.it

**Keywords:** antimicrobial resistance (AMR), cystic fibrosis, pulmonary exacerbations, CFTR channel, microbial communities

## Abstract

The phenomenon of antimicrobial resistance (AMR) is a critical global health challenge, with prospects indicating its potential to become the leading cause of death worldwide in the coming years. Individuals with pre-existing conditions, such as neoplastic disease undergoing chemotherapy, those on immunosuppressive therapy, and individuals with rare diseases like cystic fibrosis (CF), face heightened challenges due to AMR. CF is a rare disease caused by a deficiency in the synthesis of the Cystic Fibrosis Transmembrane Conductance Regulator (CFTR) channel protein, resulting in multi-organ clinical symptoms, particularly in the respiratory system. PwCF experience recurrent pulmonary exacerbations triggered by bacterial or viral infections, making them particularly vulnerable to the impact of AMR. This review delves into the complex relationship between AMR and climate dynamics, focusing on the unique challenges faced by individuals with CF. It discusses the methods employed to measure AMR, its global impact on antibiotic resistance, and the specific microbial communities present in the CF airway. The review also explores the intricacies of antimicrobial resistance within the context of cystic fibrosis, emphasizing the urgent need for research in this field.

## 1. Introduction

### 1.1. Antimicrobial Resistance (AMR)

Antimicrobials are defined as naturally or synthetic occurring pharmacological compounds that exhibit activity against disease-causing microorganisms like viruses, bacteria, parasites and fungi [1,2]. The phenomenon of antimicrobial resistance denotes the reduced effectiveness of antimicrobials against forms of pathogenic microorganisms which have developed resistance to treatment and are no longer responsive [3]. This challenge of antimicrobial resistance can be attributed to several factors, including the improper use and overuse of antimicrobials, a decline in the discovery of new antimicrobial compounds, the application of antibiotics in agriculture, and the amplified spread of resistant strains due to increased global travel. In recent years, global organizations such as the World Health Organization (WHO) have offered recommendations and proposed coordinated strategies and measures to contrast this phenomenon. However, the problem extends beyond borders, affecting countries at various stages of development, with healthcare systems facing the brunt of antibiotic shortages [4]. Microorganisms responsible for multidrug-resistant infections can develop various mechanisms to evade the lethal effects of antimicrobials [5]. In addition, the growth of disease-causing microorganisms in aggregated biofilm structures further impedes the efficacy of antimicrobial agents [6]. These aspects may have a more pronounced effect on specific diseases involving the respiratory tract, such as cystic fibrosis.

### 1.2. Global Impact of Antibiotic Resistance

Growing evidence indicates a significant correlation between AMR and adverse outcomes within healthcare settings, including heightened in-hospital mortality, prolonged length of stay, and increased healthcare costs. An economic model developed by Cassini et al. underscores the escalating and substantial burden of AMR-related infections compared to other infectious diseases [7]. A recent systematic analysis, estimating the global burden of deaths associated with AMR infections across 88 pathogen-antibiotic combinations, reported a staggering toll of 4.95 million people (95% uncertainty intervals (UI) 3.62–6.57). This figure includes 1.27 million people (95% UI 0.91–1.71) whose deaths were directly attributable to AMR [8]. The overuse and inappropriate prescribing of antibiotics contribute to the development of multidrug-resistant species in both Gram-negative and Gram-positive bacteria. In several health contexts, there is not a sufficient early identification of the microorganism causing infections and their susceptibility to antibiotics. This deficiency leads to the large use of broad-spectrum antibiotics, contributing to the development of resistant strains [9]. The advent of antibiotics has significantly transformed the dynamics of resistance. Given the propensity of bacterial species to traverse environmental and species confines, it becomes imperative to comprehend the intricate bonds within animal, human, and environmental microbiota underscoring the necessity for a comprehensive approach to cope with this global health challenge [10,11,12]. The growing threat of AMR is a matter of heightened concern, especially in less industrialized countries, where consumptions of antibiotics are on the rise.

### 1.3. The Complex Relationship between Antimicrobial Resistance and Climate Dynamics

The convergence of AMR and climate change represents a significant facet of global health. As the planet undergoes shifts in climate patterns, there is an increasing acknowledgment of the potential impact on the dynamics of antimicrobial resistance. The connection between global warming and infectious pathogens has deep historical roots [13]. For example, the adaptation of bacteria such as *Salmonella*, *Campylobacter* and *Vibrio cholerae* to higher temperatures has resulted in the resurgence of these infections [14]. Consequently, resistance rates for these bacteria are likely to rise, further limiting options for treating AMR pathogens [15,16]. As temperatures surge due to the climate crisis, AMR is on the rise across humans, animals, plants, and the environment [17,18]. In tandem, the climate change can contribute to the dissemination of novel and resurging pathogens, such as *Candida auris* and *Plasmodium falciparum* [19]. By 2050, the population at risk of vector-borne diseases is projected to reach 500 million [20]. Malaria, a predominant global vector-borne disease, is notably influenced by temperature and humidity [21]. Moreover, heat and humidity emerge as risk factors for the escalating rates of salmonellosis, that is also witnessing a rise in antibiotic resistance [22]. Similarly, infections typically associated with tropical climates, including Chagas disease, Zika, dengue, and chikungunya, are gradually shifting towards regions with higher temperatures, even during winter months [23]. Seasonality has been observed in influenza and also in several bacterial infections. For instance, bloodstream infections (BSI) [24,25,26], healthcare-associated infections (HAIs) [27], intra-abdominal, and surgical site infections [28] show a seasonal pattern, peaking during the summer [29]. The clearest evidence of this interaction is observed in the rise of vector-borne and environmentally transmitted diseases [30]. Instances such as the increased incidence of Ixodes spp. ticks, tick-borne encephalitis, and Lyme disease in northern parts of Europe and North America exemplify this correlation [31,32]. Moreover, in regions like Nepal, the emergence and upslope expansion of diseases such as dengue, chikungunya, malaria, and Japanese encephalitis, along with their vectors, underscore the far-reaching implications of climate change on infectious diseases [33]. Even in the Arctic and sub-Arctic, parasitic, vector-borne, and rodent-borne diseases like cryptosporidiosis, filariasis, tularemia, and hantavirus-induced hemorrhagic fever have surfaced due to shifting climatic conditions [34,35]. The expanding range of helminth parasites and host snails in Europe further accentuates the intricate relationship between climate change and the geographical distribution of diseases [36].

### 1.4. Fibrosis Cystic (FC)

**Figure 1:**
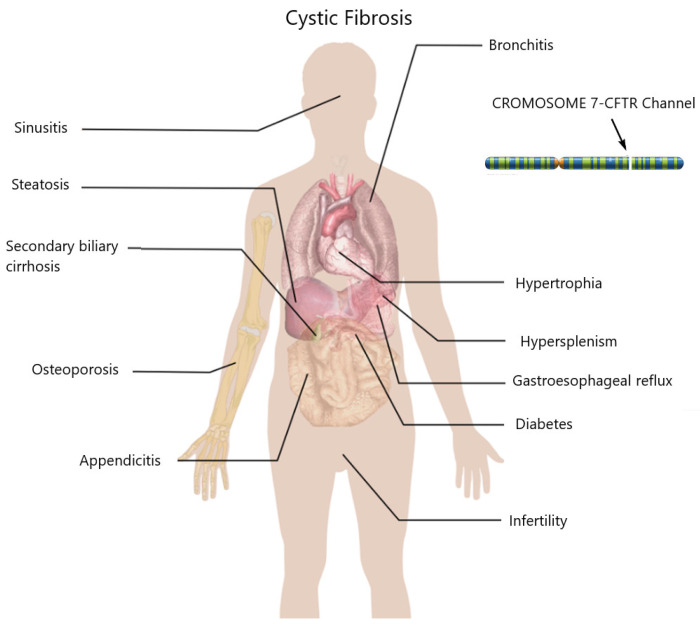
Cystic fibrosis (often abbreviated as CF, also known as mucoviscidosis or fibrocystic pancreatic disease) is a genetic disease transmitted in an autosomal recessive manner, caused by a mutation in the CFTR gene, which codes for a protein that functions as a chlorine channel called CFTR (Cystic Fibrosis Transmembrane Conductance Regulator). The clinical symptoms affect various organs, respiratory, gastrointestinal, cardiovascular, bone tissue.

Cystic fibrosis (CF) is an autosomal recessive genetic disorder, first documented by Dr. Dorothy H. Anderson in 1938, during an era when most patients did not survive beyond childhood [37]. In those years, CF patients did not survive beyond the first year of life, and it only extended to about 8 years by the 1970s [38]. The global incidence of CF is assessed to range among 1 in 2000 and 1 in 6000, with higher prevalence observed between individuals with Northern European ancestry with respect to other racial groups. Worldwide, there are over 70,000 patients living with CF (PwCF), and in the United States alone, approximately 32,000 individuals have been diagnosed, with over 1000 new cases identified annually [39]. According to the 2021 U.S. Cystic Fibrosis Foundation (CFF) Patient Registry, 91.4% of registered PwCF are of White ethnicity, 9.8% are Hispanic, 3.5% are African American, and 5.1% fall into the “other” category [40]. Notably, PwCF over 18 years old is on the rise and has surpassed PwCF under 18 years old, with approximately 58% of individuals with CF in the United States being 18 years and older. Cystic fibrosis is caused by a mutation in the CFTR gene, which codes for a protein that functions as a chlorine channel called CFTR (Cystic Fibrosis Transmembrane Conductance Regulator). The lack of CFTR channel activity causes abnormal fluid consistency that can lead to inflammation, pulmonary infection with disrupted mucociliary transport and lung damage, and eventually progressive multi-organ dysfunction (Figure 1) [41].

PwCF commonly face the progression of respiratory disease, potentially leading to respiratory failure and an elevated risk of having pulmonary hypertension (PH) and consequent right ventricular dysfunction. Set of symptoms which affects various internal organs can be attributed to the malfunction in the excretion of chloride. This anomaly results in the production of abnormally thick and viscous mucus. Consequently, the blockage of major passages leads to significant respiratory symptoms, including recurrent lung infections known as pulmonary exacerbations. These exacerbations and the ensuing lung damage predominantly stem from frequent infections. In CF individuals, the airways undergo microbial colonization shortly after birth, with peripheral lung settlement and chronic phlogosis triggering afterward [42]. Differently from lung infections in non-PwCF, CF-related infections tend to persist and lead to phenotypic changes in the infecting organisms [43]. The respiratory route more obstructed is the pulmonary tract, because of the partial hypersecretion of mucus from the submucous glands. Furthermore, the presence of chronic or acute inflammation often leads to the collapse of the UA during sleep, which can trigger obstructive sleep apnea (OSA), a disorder that manifests itself with intermittent hypoxia and creates disturbances in the normal sleep cycle [44]. Research indicates a close association between OSA and structural alterations in the oropharynx, as well as chronic rhinosinusitis. OSA is strongly linked to coronary artery disease (CAD), heart failure, hypertension, and cardiac arrhythmias. Over the course of their lives, individuals with CF undergo repeated antibiotic treatments, particularly during disease exacerbations. The combination of antibiotic administration and emerging therapeutic approaches, like mutation-specific modulator treatments, has significantly extended life duration. This extension is primarily attributed to the management of respiratory complications. On the other hand, growing evidence suggests that existing therapeutic strategies often fall short of completely eradicating involved pathogens. Standard dosing regimens may lead to sub-therapeutic antibiotics levels, thereby raising the risk of the failure of the treatment and, more critically, contributing to the emergence of AMR [45]. The widespread alternation between ‘suppressive’ and ‘curative’ antibiotic schemes further heightens the AMR’s risk [46]. Common micropathogens causing lung infections in PwCF include *Staphylococcus aureus (SA)*, *Haemophilus influenzae*, and *Pseudomonas aeruginosa* [47]. Alongside bacterial infections, viral and fungal infections, such as *Aspergillus fumigatus*, are also typical in these cases [48]. Another concerning condition is the infection caused by the *Mycobacterium avium* complex (MAC), a group of bacteria similar to tuberculosis. These bacteria can lead to lung damage and are not responsive to standard antimicrobial treatments. Addressing these infections typically involves the use of antimicrobial chemotherapy agents. As a result, the challenge of antibiotic resistance becomes a particularly consequential issue for individuals dealing with cystic fibrosis.

## 2. Discussion

### 2.1. Antimicrobial Resistance in Cystic Fibrosis

**Figure 2:**
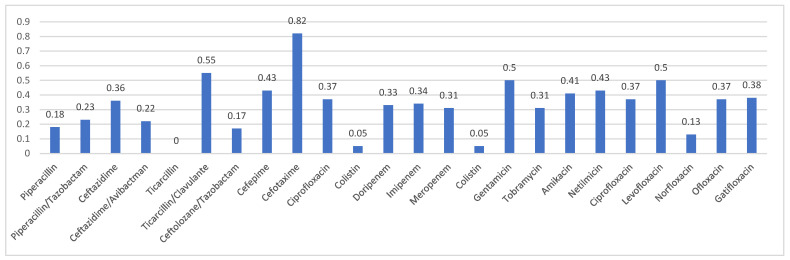
The graph shows the prevalence of weighted resistance of *Pseudomonas aeruginosa* to the various antibiotic classes over the period 2011–2021 [59].

People with CF are more susceptible to microbial infections. Several pathogenic microorganisms such as *Pseudomonas aeruginosa*, methicillin-resistant *Staphylococcus aureus* and *Burkholderia* genus species are associated with worse lung function. In many cases, antimicrobial agents are administered on a daily basis to contrast microbial infections, to treat pulmonary damages or for maintaining suppressive therapy in the cases of chronic infections, either on an inpatient (i.e., intravenous) or outpatient (i.e., inhaled or oral) basis. The usage of antimicrobials unavoidably triggers concerns regarding the emergence of increasingly resilient pathogenic microorganisms. Consequently, antimicrobial resistance has become a well-recognized and escalating issue in the context of Cystic Fibrosis. Moreover, the challenge of AMR in PwCF is exacerbated by the creation of microbial clusters within an extracellular matrix, impeding the effectiveness of common antimicrobials in reaching their intended targets. These clusters are termed biofilms. Biofilms are intricate and organized structures formed by bacteria, surrounded by a matrix of various macromolecules. This matrix creates a protective barrier, providing pathogens with defense against changing environmental conditions [49]. The formation of these biofilms appears to be triggered by quorum-sensing signals extracellular chemical signals that regulate gene expression in a density-dependent manner [50]. In the context of CF, biofilms create a microenvironment that supports the maturation and protection of bacteria and viruses, making them resistant to both antibiotics and the host’s immune mechanisms. The tolerance of biofilms extends to numerous biological processes, and this tolerance increases as the biofilm matures. The presence of biofilms leads to prolonged pulmonary contagions in individuals with CF, lastly leading to the insurgence of respiratory complications [51]. Recent searches utilizing culture-independent molecular techniques have revealed the intricate dynamics of the airway microbiome in CF, involving not only the ‘traditional’ pathogens identified but also other atypical microorganisms [52]. However, antibiotics face challenges in eradicating pathogens that form biofilms. This is primarily due to the inherent antibiotic tolerance of biofilm-forming pathogens and, secondarily, because biofilms facilitate the contingency and spread of mutational antibiotic resistance. The biofilm resistance to antibiotic agents is complex and is traceable to physical, physiological, and genetic factors. In contrast, AMR arises from mutations that occur following repeated exposure to high concentrations of antibiotics [53]. The development of biofilms either shields bacteria from the host immune system and antibiotics, or enables their proliferation in an environment with low oxygen levels and limited nutrients. Biofilms consist of numerous bacterial sub-communities exhibiting varying degrees of metabolic activity. Peripheral sub-populations display high metabolic activity, consuming significant amounts of oxygen and nutrients. In contrast, sub-populations located in the inner layers exhibit lower or zero metabolic activity, making them more tolerant to antimicrobial agents and contributing to the persistence or recurrence of infections [54]. To address biofilm-related infections, higher concentrations of antibiotics or liposomal antibiotic formulations can have more efficacy. However, this comes at the cost of an increased risk either of toxicity or the development of AMR [55]. Biofilms are now recognized as a pivotal factor in prolonged and recurrent pulmonary infections in PwCF. Notably, newly formed biofilms are more susceptible to antibiotic agents compared to the olders, underscoring the importance of prompt and well-designed therapeutic strategies [56,57]. Multiple indications point to the presence of *Pseudomonas aeruginosa* in biofilm form within the deep airways of individuals with CF, clarifying the difficulties in eradicating these bacterial infections [58]. The Figure 2 illustrates data prevalence of weighted resistance to antibiotic classes [59].

### 2.2. Microbial Communities in the Cystic Fibrosis Airway

The microbiota within the airways of individuals with cystic fibrosis (CF) exhibits significant diversity among different individuals [60]. In PwCF, there is a distinct pattern of bacterial species in the nasopharynx, primarily characterized by a high concentration of *SA* [61]. This pattern tends to establish itself early in life, suggesting alterations in the small environment of the nose that facilitate the settlement by *SA* whereas hindering the proliferation of pathogens. As a result, the nasal cavity may serve as an indicator of the starting of *SA* settlement in the lower pulmunoray area, particularly in the years of childhood [62]. Within the realm of viral infections, epidemiological data demonstrate an overall prevalence of pulmonary infections caused by viral agents in CF individuals ranging between 13 and 60 percent [63]. Among the most frequently identified viral infections are respiratory syncytial virus (RSV) and human rhinovirus. As for fungal infections, *Aspergillus* species, with prevalence rates reaching 78%, are the most commonly detected. Occasionally, *Aspergillus* infections can lead to bronchitis accompanied by lung inflammation. Consequently, the CF airways comprehends a variety of physiochemical and nutritional microenvironments that play a role in guiding pathogen interactions and contributing to the pathogenesis of the disease. The lack of bicarbonate ion secretion due to the CFTR channel’s functional deficiency results in thicker, more viscous mucus and a lower pH microenvironment. This affects the antibiotics’ ability to hinder bacterial growth. Moreover, robust oxygen gradients and anaerobic conditions may form in CF individual airways, making certain bacterial species more resistant to common antibiotics. While early infections in PwCF can be effectively eradicated with appropriate antibiotic treatment, the prevalence of protracted and chronic infections tends to increase with age. Upon recognizing a particular pathogen for the first time, it is essential to promptly and vigorously apply antibiotics to eradicate the microorganism. This helps prevent persistent settlement and alleviates potential long-term negative outcomes. Nevertheless, the ever-changing characteristics of the airway microenvironment, combined with the variety of pathogens present, especially in prolonged contagions, lead to a disparity between the effectiveness of antibiotics observed in laboratory settings and their performance in living organisms. This discordance, in turn, leads to an elevated failure rate of antimicrobial treatment. The airway microenvironment undergoes continuous changes, and chronic infections often involve a multitude of pathogens. This complexity may account for the challenges in achieving consistent treatment outcomes. The discrepancy between laboratory studies (in vitro) and real-world conditions (in vivo) underscores the need for a more comprehensive understanding of the intricate interactions within the CF airways. The comprehensive management of CF infections requires a nuanced approach, considering the evolving nature of the microbial landscape in the airways and the intricate dynamics that contribute to the success or failure of antimicrobial interventions.

### 2.3. The Role of CFTR Modulators on Microbial Communities in Cystic Fibrosis Airways

The pathophysiology and molecular mechanisms linking genetic dysfunction of the CFTR channel protein to bacterial infections and chronic airway inflammation are not yet totally clarified. As previously described, CFTR impairment leads to a dysfunction of the environment of the respiratory tract. In recent years, new drug therapies aimed at correcting the underlying defect, modulators of the CFTR channel protein, have come onto the market, leading to a substantial clinical improvement in PwCF, modifying disease progression [64,65]. Evidence shows that restoration of CFTR channel protein function is associated with improved mucociliary clearance, and airway surface hydration, enhancing endogenous defence mechanisms by influencing the microbiology and response to bacterial infections in CF airways. CFTR is a channel that influences fluid and pH in the surface fluid of the airways. Studies in CF pigs have demonstrated impaired bacterial killing and disruption of mucociliary transport, resulting in infections and mucus obstruction [66]. However, despite the consolidated evidence supporting these pathophysiological mechanisms, given the complexity of this pathology, further studies are underway to demonstrate the role of CFTR modulators in reducing the long-term incidence and prevalence of chronic airway infections. In this regard, some evidence has shown that CFTR modulators are able to reduce the prevalence of bacteria, in particular the key pathogen of CF, *Pseudomonas aeruginosa* (*P. aeruginosa*), in treated patients. In addition, several in vitro studies highlight a synergistic interaction between CFTR modulators and specific antibiotics, with indirect results in counteracting AMR. Certain searches have demonstrated that with disease advancement, the diversity of the pulmonary microbiome in individuals with CF decreases, with opportunistic pathogens, such as *P. aeruginosa*, predominating [67] Evaluation of changes in the airway microbiome in response to CFTR modulators is thus crucial to improve therapeutic strategies for the management of airway infections [68]. Studies investigating the composition of the lung microbiota in individuals with CF before and after treatment with modulated VAT have shown a shift towards a ‘healthier’ community composition of microorganisms, which correlates with the levels of circulating inflammatory markers [69]. Further evidence has shown that the addition of CFTR channel modulators induces an increase in microbial diversity in the airways of PwCF [70], but the underlying mechanism is not fully understood to date. Recent studies have also assessed the potential synergistic effects of CFTR modulators with antimicrobial chemotherapeutics. In vitro studies suggest also bactericidal and bacteriostatic activity to the CFTR modulators against SA and *P. aeruginosa* [71]. Further studies are needed to fully understand the role of CFTR modulators in bacterial infections and contrast to AMR in individuals with FC [72,73].

### 2.4. Most Clinically Relevant Pathogens

#### 2.4.1. *Pseudomonas aeruginosa*

*P. aeruginosa* is recognized as an exceptionally important opportunistic pathogen in humans, renowned for its impressive ability to exhibit various phenotypes and adapt effectively [72]. It takes a central role as the leading cause of chronic lung infections in PwCF, with its prevalence escalating with age from 10 to 30% in preschoolers to over 80% in young adults [73,74]. In younger patients, colonization often involves multiple wild-type, non-mucoid strains, which progressively transform into mucoid variants, forming biofilms. The intrinsic ability of *P. aeruginosa* to adapt to diverse environments underlies its capacity to induce a range of acute and chronic infections. The prolonged settlement of the airways is a defining feature of CF and is associated with increased morbidity and mortality. The typical onset of colonization is observed among 6.5 and 7.1 years [75]. Early infection with *P. aeruginosa* may serve as an indicator of premature decline in lung function, underscoring the importance of proactive monitoring in individuals with early disease onset [76]. Notably, despite aggressive antibiotic treatment, *P. aeruginosa* often persists in the airways of PwCF. Although early eradication with antibiotics is possible, intermittent and chronic infections become more prevalent as individuals age. The shift from intermittent to chronic infections is primarily linked to *P. aeruginosa* tendency to form biofilms [77]. The onset of antimicrobial resistance in the context of *P. aeruginosa* is not only associated to biofilm development; in fact, the pathogen demonstrates adaptability to its environment through some virulence factors and the creation of multi-drug-tolerant persister cells, dormant variants that randomly emerge in microbial populations [78]. These mechanisms often operate simultaneously, leading to resistance against nearly all available antibiotics. The combination of *P. aeruginosa*’s virulence and its heightened capacity for AMR contributes to the complexity of lung infections in PwCF, making them challenging to treat [79]. In CF management, antibiotics play a pivotal role. In particular, antibiotics are employed to eliminate *P. aeruginosa* during the early phases of settlement, manage respiratory impairments, and manage relapsing or chronical infections [80]. Setting the correct antibiotic therapy based on in vitro tests is essential to combat *P. aeruginosa* and to prevent complications in affected individuals. However, although antibiotic treatment can be effective in reducing the bacterial load, it is often difficult to completely eradicate the infection [81]. Literature data underscore that *P. aeruginosa* species isolated from PwCF display higher resistance to commonly used antibiotics like ciprofloxacin, tobramycin, and meropenem compared to isolates from non-PwCF [82]. Presently, multidrug-resistant *P. aeruginosa* infections pose a significant health issue in PwCF, making long-term disease management particularly challenging. In this context, timely and deepened knowledge of *P. aeruginosa* AMR patterns at the level of infected people becomes essential.

#### 2.4.2. *Staphylococcus aureus*

*Staphylococcus aureus* is one of the initial bacteria to settle the airways in individuals with CF. Consequently, its prevalence is higher in the initial stages of the disease, exceeding 50% in infants and peaking at around 80% in early adolescence. Moreover, several *SA* species resistant to key antibiotics have been identified in PwCF, and their presence correlates with rapid respiratory function decline and poorer clinical outcomes. *SA* faces the ongoing challenge of adapting to the adverse conditions within the respiratory tract, contending with host immunological responses, antibiotic exposure, and competition with other pathogens [83]. Additionally, *SA* grows through biofilm, further contributing to develop AMR. In young people with CF, the presence of *SA* is associated with significant phlogosis of airways and more grave pathology [84]. Methicillin-resistant *SA* (MRSA) poses a distinct threat to PwCF, generating lung impairment and increasing hospitalization rates. Furthermore, MRSA serves as a catalyst for the failure to maintain pre-infection pulmonary functionality, even following the administration of parenteral antibiotic agents for pulmonary impairments. However, the accord on the best management of both MRSA and methicillin-sensitive *SA* (MSSA) is currently lacking [85,86]. Consequently, the primary goal remains attaining the best care, with early eradication being advisable. Research that studies the early onset of treatment, immediately after having positive culture results for MRSA, is essential to guide the management of therapy at a clinical level, particularly among subjects without symptoms [87]. While *SA* and *Pseudomonas aeruginosa* stand out as the main bacteria in CF, the latter tends to replace the former through the course of the illness [88,89]. Interestingly, only *Pseudomonas aeruginosa* strains noticed in premature infections appear to rival with *SA*. In contrast, those isolated from chronic infections exhibit less antagonistic behavior, suggesting that these two pathogens are capable of interacting in vivo [90]. In fact, many PwCF with co-infections experience a synergistic interaction, increasing rates of respiratory impairments.

#### 2.4.3. *Burkholderia Genus*

The *Burkholderia* genus, originally known as *Pseudomonas cepacia*, constitutes a group of aerobic, catalase and oxidase-positive, gram-negative bacteria encompassing 22 closely related strains [91]. The most frequently isolated species include *Burkholderia cenocepacia*, *Burkholderia multivorans*, and *Burkholderia cepacia*. These species are nearly indistinguishable phenotypically and can only be differentiated through genetic or biochemical characteristics [92]. Typically found in soil or water sources [93], their reservoirs remain unknown for some of them. Bacteria within the *Burkholderia* genus complex are recognized as prominent opportunistic pathogens, particularly in PwCF. In cystic fibrosis, *Burkholderia cenocepacia* and *Burkholderia multivorans* account for 85% to 97% of *Burkholderia* genus complex infections, whereas *Burkholderia cepacia* is more prevalent in non-PwCF. These bacteria are associated with quick declines in respiratory system and the occurrence of the ‘cepacia syndrome’, characterized by necrotizing pneumonia and uncontrolled deterioration leading to septicemia and often death [94]. Patients infected with *Burkholderia cenocepacia* show worse outcomes after lung transplantation, leading many healthcare facilities to remove them from the transplant list. Bacteria within the *Burkholderia* genus complex inherently exhibit resistance to numerous antibiotics, including penicillins, cephalosporins, and aminoglycosides. While the potential for these infections to become fatal necessitates careful management, there is currently a lack of comprehensive evidence to establish tools to contrast *Burkholderia* genus infections affecting PwCF. Consequently, the treatment of these infections primarily relies on in vitro information and individual susceptibilities.

#### 2.4.4. *Achromobacter*

*Achromobacter* bacteria are characterized as motile, gram-negative, and exhibit positive oxidase and catalase reactions. Hospital outbreaks often stem from contaminated solutions. *Achromobacter* strains are frequently identified in all the respiratory tracts of PwCF [95,96]. These bacteria are commonly found in watery habitats, and may are present in health facilities. These bacteria typically display resistance to antibiotics, employing complex AMR mechanisms that currently are not fully clarified [97]. Chronic colonization by *Achromobacter xylosoxidans* in PwCF is traced back to a clear decrease in lung function [97]. The study by Somayaji et al. on 1103 cystic fibrosis patients over 18 years found that chronic *Achromobacter* infection increased the hazard of passing or graft. Although patients with chronic infection had more pulmonary exacerbations, this effect became non-significant after adjustments. The chronic infection group had lower lung function (FEV1%), but lung function did not worsen after developing chronic infection. Overall, chronic *Achromobacter* infection in cystic fibrosis is combined with an elevated risk of unfavorable effects. Currently, there are considerable challenges in the laboratory diagnosis of *Achromobacter* species, primarily due to incorrect identification with other bacteria strains. As a result, *Achromobacter* species have garnered increased research attention in recent years [98].

## 3. Conclusions

The escalating global issue of antibiotic resistance (AMR) necessitates urgent attention and a paradigm shift in antibiotic usage. Without collective commitment and a comprehensive approach, the fate of new antibiotics may mimic that of their predecessors, succumbing to ineffectiveness against evolving drug resistance. Mitigating the impact of AMR requires improvements in antimicrobial stewardship, enhanced diagnostics, and stringent infection control measures. Global collaboration is essential to combat the emergence and spread of untreatable infections. The intricate link between AMR and climate change emphasizes the need for a holistic approach to address these interconnected challenges. Recognizing the interplay between environmental factors, microbial ecosystems, and human health is crucial for developing effective strategies to mitigate the impact of both antimicrobial resistance and climate change on global well-being. In the context of cystic fibrosis (CF), AMR poses a persistent challenge. Advances in molecular technologies, particularly whole-genome sequencing (WGS) from next-generation sequencing (NGS) platforms, offer new prospects for genotypic recognition of bacterial resistance profiles. This method provides greater discriminatory ability and efficiency, especially in detecting acquired resistance genes and chromosomal mutations in polymicrobial communities within the airways of PwCF. However, research gaps still exist, requiring further elucidation of factors such as virulence, mechanisms of AMR, systematic treatment guidelines, and the need for more studies in children to address pharmacokinetic differences. The complexity of interactions between different species and the different facets of the respiratory pathophysiology of CF represent challenges for the development of new effective prevention and therapeutic tools. The research aims to create new and improved diagnostic solutions, such as metagenomic sequencing and next-generation transcriptomics, for the direct detection of antimicrobial resistance in microbial communities plaguing the airways of cystic fibrosis patients.

## Data Availability

Full availability of data and materials. All stated data can be provided on request to the reader.
